# Feasibility and Acceptability of the Aboriginal and Islander Mental Health Initiative for Youth App: Nonrandomized Pilot With First Nations Young People

**DOI:** 10.2196/40111

**Published:** 2023-06-07

**Authors:** Kylie M Dingwall, Josie Povey, Michelle Sweet, Jaylene Friel, Fiona Shand, Nickolai Titov, Julia Wormer, Tamoor Mirza, Tricia Nagel

**Affiliations:** 1 Menzies School of Health Research Charles Darwin University Alice Springs Australia; 2 Menzies School of Health Research Charles Darwin University Darwin Australia; 3 Menzies School of Health Research Charles Darwin University Adelaide Australia; 4 Black Dog Institute University of New South Wales Sydney Australia; 5 MindSpot Clinic Macquarie University Sydney Australia; 6 headspace Darwin Anglicare NT Darwin Australia

**Keywords:** digital mental health, First Nations, Indigenous, young people, feasibility study, digital health, mental health, depression, mHealth, mobile app, aboriginal, acceptibility, youth

## Abstract

**Background:**

Despite young First Nations Australians being typically healthy, happy, and connected to family and culture, high rates of emotional distress, suicide, and self-harm are also observed. Differing worldviews of service providers and First Nations young people regarding illness and treatment practices, language differences, culturally inappropriate service models, geographical remoteness, and stigma can all inhibit access to appropriate mental health support. Mental health treatments delivered digitally (digital mental health; dMH) offer flexible access to evidence-based, nonstigmatizing, low-cost treatment and early intervention on a broad scale. There is a rapidly growing use and acceptance of these technologies among young First Nations people.

**Objective:**

The objective was to assess the feasibility, acceptability, and use of the newly developed Aboriginal and Islander Mental Health Initiative for Youth (AIMhi-Y) app and determine the feasibility of study procedures in preparation for future assessments of effectiveness.

**Methods:**

This was a nonrandomized pre-post study using mixed methods. First Nations young people aged 12-25 years who provided consent (with parental consent where appropriate) and possessed the ability to navigate a simple app with basic English literacy were included. Researchers conducted one face-to-face 20-minute session with participants to introduce and orient them to the AIMhi-Y app. The app integrates culturally adapted low-intensity cognitive behavioral therapy (CBT), psychoeducation, and mindfulness-based activities. Participants received supportive text messages weekly throughout the 4-week intervention period and completed assessments of psychological distress, depression, anxiety, substance misuse, help-seeking, service use, and parent-rated strengths and difficulties at baseline and 4 weeks. Qualitative interviews and rating scales were completed at 4 weeks to gain feedback on subjective experience, look and style, content, overall rating, check-ins, and involvement in the study. App use data were collected.

**Results:**

Thirty young people (17 males and 13 females) aged between 12 and 18 (mean 14.0, SD 1.55) years were assessed at baseline and 4 weeks. Repeated measures 2-tailed *t* tests showed improvements in well-being measures that were statistically and clinically significant for psychological distress (Kessler Psychological Distress Scale, 10-item) and depressive symptoms (Patient Health Questionnaire, 2-item). Participants spent on average 37 minutes in the app. The app was rated positively, with mean ratings of 4 out of 5 points (on scales of 1-5). Participants reported that they found the app easy to use, culturally relevant, and useful. The feasibility of the study was demonstrated with a 62% recruitment rate, a 90% retention rate, and high study acceptability ratings.

**Conclusions:**

This study supports earlier research suggesting that dMH apps that are appropriately designed with and for the target populations are a feasible and acceptable means of lowering symptoms for mental health disorders among First Nations youth.

## Introduction

Young First Nations Australians are typically healthy, happy, and connected to family and culture [1,2]. A 2017 national survey revealed 76% of young First Nations Australians reported feeling happy most or all of the time, 73% felt they were able to have a say on important issues within their family, and 59% had very positive or positive feelings about the future [2]. Nevertheless, rates of emotional distress are high, with around one-third of First Nations’ young people experiencing high to very high levels of distress, 2 in 5 experiencing mental health conditions, and suicide being a leading contributor to the disease burden [2]. Despite the high need, First Nations young people are particularly reluctant to seek help [2]. Constraints include stigma, discrimination, differing worldviews, language differences, cost, and transport issues [3,4]. Adolescence is a particularly prime time for intervention, as approximately 50% of mental illness emerges during that period [5]. Implementation of successful prevention efforts early is more likely to prevent the onset of mental disorders [5]. Timely access to culturally appropriate, effective prevention and treatment for First Nations young people is therefore imperative.

There is increasing recognition of the potential for digital mental health (dMH) interventions to overcome access issues and address the unique and significant mental health needs of First Nations people in rural and remote areas [6]. The potential benefits of digital health solutions, particularly those developed and implemented in a culturally informed way, could be substantial [7]. There are several ways dMH tools can be used. They can be used as a stand-alone treatment or augment existing treatments through blended care or fully supported treatments [8]. One particular benefit is that they can extend therapeutic activities such as assessments, monitoring, support, and interventions into real-world settings [9,10]. In the First Nations context, dMH tools developed in a culturally responsible and appropriate manner have shown great potential in supporting the development, maintenance, and strengthening of First Nations cultural identity [11].

While there are many apps available, there is limited evidence base for the majority of them. A recent study found over 1500 apps publicly available for addressing depressive symptoms; however, only 32 studies were presented in the academic literature [12]. For young First Nations Australians, and particularly those in remote regions, there are limited culturally specific dMH options. A recent review identified 3 individual-directed web-based apps available in the Australian First Nations context [7]. These were MindSpot, iBobbly, and Aboriginal and Islander Mental Health Initiative’s (AIMhi) Stay Strong, each with some level of evidence for their acceptability and effectiveness [6,13-17].

In Australia, recent randomized controlled trials (RCTs) have promoted culturally adapted mental health apps as a feasible and acceptable means of lowering symptoms of mental illness for First Nations young people [16] and adults [14]. The iBobbly app has shown promise for reducing risk factors for suicide among First Nations young people through the use of acceptance and commitment therapy, mindfulness, and self-soothing activities [15,16]. Similarly, the AIMhi Stay Strong app (and the paper-based Stay Strong Plan) is a culturally adapted motivational care planning (MCP) therapy that has demonstrated effectiveness in reducing psychological distress, depressive symptoms, and substance misuse among First Nations adults [14,18]. This therapy uses a holistic and strength-based approach consistent with First Nations conceptualizations of social and emotional well-being [19] and incorporates culturally adapted low-intensity cognitive behavioral therapy (CBT) and motivational interviewing elements. The Stay Strong therapy adopts an empowering, person-centered perspective, incorporating the 4 steps of identifying supportive people, strengths, worries, and setting goals for change.

While effectiveness is generally assessed through an RCT, evaluation of the feasibility, acceptability, and engagement with dMH interventions requires varied methodologies, and a combination of both subjective and objective criteria are suggested as appropriate for understanding user engagement [20]. For example, feasibility can be assessed through recruitment, retention, adherence, and completion rates. Acceptability and engagement are often measured through the extent of use (eg, app opens, minutes spent in the app), along with user-reported subjective views and experiences [21,22]. Given the speed at which technologies advance, the methods for evaluating dMH tools need to be flexible and proportionate to the tools’ complexity and the anticipated size of the effect on users’ mental health [21,22]. The time and resources required for planning and undertaking an RCT are not always appropriate for the assessment of dMH tools due to their rapid development and the accelerating time to the obsolescence of technological innovations [9,22,23]. There is a need for better-quality early evaluation of new digital health products, whether an initial summative evaluation of an established product to help decide whether it is worth adopting or a formative evaluation of an app during its development [22].

In 2020, three years of co-design workshops with First Nations youth resulted in the development of the first version of a new mental health app, the AIMhi for Youth (AIMhi-Y) app [24,25]. The participatory design process identified young First Nations Australians’ lived experiences of mental health and well-being and the dMH tool features preferred by young people and service providers. It also assessed the alignment of these preferences with recommendations from the scientific literature (including CBT, behavioral activation techniques, self-monitoring, notifications, gamification, etc) to design the new app [24,25]. The aim of this study was to assess the feasibility, acceptability, and use of the newly developed AIMhi-Y app and to determine study feasibility in preparation for future assessments of effectiveness.

## Methods

### Study Design

**Figure 1:**
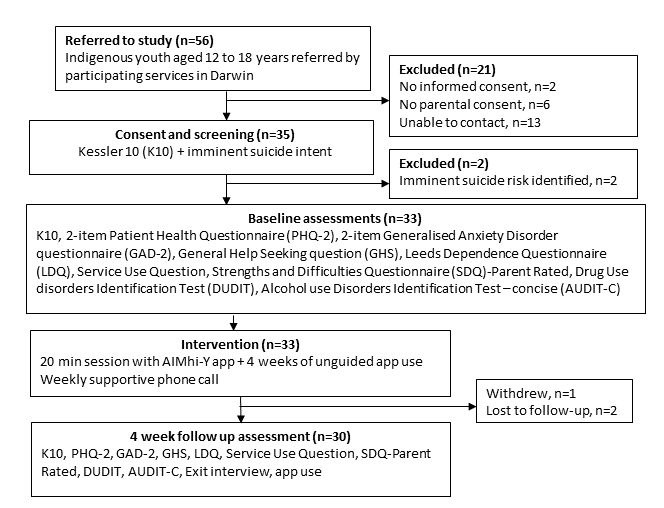
Participant flow through the study.

This is a nonrandomized pilot study using mixed methods to assess the newly developed AIMhi-Y app. The feasibility of conducting a larger-scale trial was tested using an uncontrolled single-group prepost design. The focus was on assessing the feasibility of the study methods, app implementation, app user engagement, and outcome measures, as well as gaining feedback on the barriers and acceptability of the app and study methods. The intervention period was 4 weeks. Data collection included prepost-delivery of outcome assessments at baseline and 4 weeks, researcher observation during intervention delivery, completion of qualitative exit interviews including app and study ratings at 4 weeks, and review of app use data (see Figure 1).

### Participants and Setting

Participants were a convenience sample of young people referred to the study from participating services in Darwin, Northern Territory (NT), including Anglicare NT headspace, Stars Foundation, Clontarf Foundation, and the Council for Aboriginal Alcohol Program Services. Eligible participants were those identified by key contacts at each organization as likely to benefit from an introduction to a well-being app. They included First Nations young people aged 12-25 years with basic English literacy and the ability to navigate a simple app, for whom informed consent was gained from the individual and their parent or guardian.

### Governance

An Indigenous Youth Reference Group (IYRG) involving 21 young people (86% female), 15-25 years old, residing in regional (n=11), remote (n=5), or very remote (n=5) settings, met (in person or via videoconference) 4 times over the study period. They provided advice regarding study procedures, the interpretation of data, and ideas for future app iterations. Meetings lasted 1.5-2 hours and were facilitated by a First Nations youth researcher and a nonindigenous project manager. Twelve IYRG members had been involved in the earlier co-design and development of the AIMhi-Y app [24,25].

An Expert Reference Group (ERG) met 3 times, gathering 10 youth service and research professionals (external to the Menzies research team; 4 of whom were First Nations Australians). The ERG assisted in refining study procedures and interpreting and disseminating findings in an advisory capacity.

The Menzies research team made key decisions with advice from the IYRG and ERG and carried out oversight of the project and the day-to-day tasks of the research. It included 3 First Nations youth researchers studying vocational education and training certificates in community health research, community services, or business, a senior cultural advisor and Larrakia Nation traditional owner, as well as a nonindigenous project manager and 3 nonindigenous senior researchers. The First Nations youth researchers were trained in study procedures and supported by the project manager with clinical mental health experience. Although involved throughout all stages of the study, the youth researchers played a particularly key role in refining study procedures and leading engagement, consent, and data collection with the young people.

### Ethics Approval

Ethical approval was obtained from the Menzies School of Health Research Human Research Ethics Committee (HREC 2020-3851), including the Aboriginal subcommittee and the Northern Territory Department of Education Research Ethics Committee (reference number 16287). We endeavored to achieve Indigenous data sovereignty through the review and approval of study design by the Aboriginal Ethics Subcommittee and engagement with the First Nations research team and Reference Group members in the co-design of data collection tools, data collection, and interpretation of data. Participants could control the data they inputted into the app and choose to maintain or delete it postproject.

### Referral and Screening

Eligible young people were referred through key staff at each organization. Young people’s parents or guardians (if under 16 years old or attending school) were contacted by a research officer via telephone or face-to-face to gain informed (verbal) consent prior to contacting and gaining informed consent from young people. At the suggestion of the IYRG, an additional “study agreement” was included to ensure participants were aware of the expectations of them while in the study (ie, to contact a support person if distressed and to use the app at some point in the following 4 weeks in their own time so they could provide feedback). Referred young people were screened prior to entering the study using the Kessler Psychological Distress Scale (K10) to test the feasibility of the screening process (for a later efficacy study, which would potentially exclude those likely to be well (K10 score <20). However, those with K10 scores <10 remained in this study as the main focus was to gather feasibility and acceptability data. K10 is sensitive to symptoms of both anxiety and depression and has been extensively used in state and national First Nations surveys [26,27]. To ensure safety, young people were also screened for imminent suicidal intent using the adapted PHQ-9 suicide item: “Have you been thinking about hurting yourself or killing yourself?”; as well as with follow-up questions: “Have you EVER, in your WHOLE LIFE, tried to kill yourself or made a suicide attempt?” (from the PHQ-9 modified for teens [28]) and “Have you been thinking about hurting yourself or killing yourself TODAY?” Those answering yes to either follow-up question were excluded from the study and referred to the immediate care of the person identified as the organization’s key study contact with a recommendation for urgent mental health assessment. At the request of the Anglicare NT headspace site, the exclusion criteria were relaxed at that site only to include individuals with suicidal intent and those with symptoms of early psychosis. The Anglicare NT headspace team was keen to offer the service to this broader client group and was confident that they were otherwise well supported within their service.

### Participant Demographics and Flow

There were 56 young people referred (with 43 able to be contacted), of whom 35 consented and were screened to enter the study (see Figure 1). Of the 35 participants, 2 were excluded due to imminent suicidal intent and referred to the site contact for follow-up. Two were unable to be followed up on, and one withdrew. Data were analyzed from 30 participants (17 males and 13 females) aged between 12 and 18 (mean 14.0, SD 1.55) years. The majority (n=25) spoke English at home (one also spoke Wadja, another also spoke Kriol), and 3 spoke Yolngu Matha at home (2 not reported). The majority usually resided in Darwin, while 2 resided in a remote community (3 were not reported).

### Intervention

**Figure 2:**
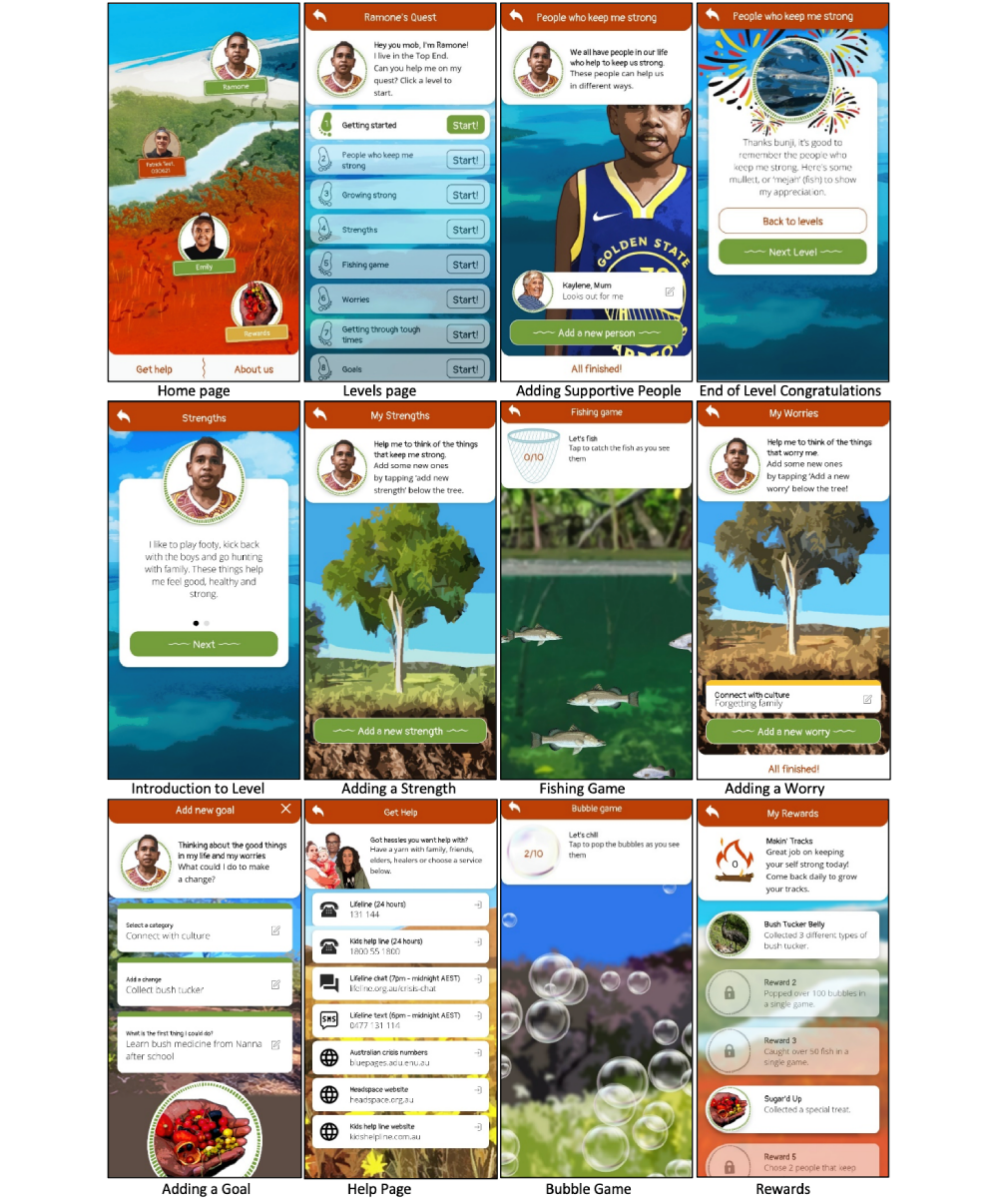
Various screenshots from the Aboriginal and Islander Mental Health Initiative for Youth app.

The smartphone-based AIMhi-Y app (version 1.0) integrates culturally adapted low-intensity CBT, psychoeducation, and mindfulness-based activities [24]. Participants begin by assisting fictional characters through a series of levels of a “quest,” aiming to become familiar with the content before beginning their own quest (ie, inputting their own information). Each quest presents 9-10 levels, which include the 4-step AIMhi Stay Strong MCP therapy. This involves the identification of (1) supportive people, (2) strengths, (3) worries, and (4) lifestyle changes or goals, which are interspersed with psychoeducational videos and games (as separate levels) [13,18,24,25]. Activities and information target both anxiety and low mood. For example, videos describe the app and getting started; the tree metaphor for growing strong; tips for how one of the characters uses mindfulness principles to find his calm; and 4 simple steps users might do to get through tough times, such as talking with a trusted person, doing more of the things that keep them strong, doing less of the things that take their strength away, or getting help from a health service. A summary page collates user or character information and presents progress. Mini-games were included in an attempt to promote relaxation, encourage real-world activities, and provide fun, engaging, and immersive sensory experiences [29]. For example, the bubble game invites users to pop bubbles as they float across the page while calming music is played; the fishing game uses a similar concept to “catch” fish; and the animal game invites users to imagine they are on a bush walk and asks them to listen to animal sounds and identify the animal. The app is easy to use with simple and intuitive designs and nonclinical, youth-friendly language. Aboriginal language words are interspersed throughout the stories, relevant to specific characters, but the majority is in English given the large number of Aboriginal languages spoken in the region. Users can select from the options presented, edit them, or input their own text at each of the 4 steps. Vibrant colors and design elements reflect the natural landscapes of different NT regions (see Figure 2). The app can be used offline, but a web-based database collates app use statistics once reconnected to the internet.

Upon initial contact, researchers conducted a face-to-face, 20-minute session with participants to introduce and orient them to the app. After commencing one of the 2 character quests (ie, Ramone or Emily) and reviewing progress made, participants were then encouraged to take the app away and complete that character quest as well as their own quest (ie, input their own supportive people, strengths, worries, and goals) prior to the next appointment at 4 weeks. Participants received a standardized supportive text message weekly throughout the 4-week intervention period. These texts were to provide low-key well-being support, encourage app use, and troubleshoot any other issues with app access. For example, “Hi [name], [researcher’s name] here just wondering how you are going. Can you give us a call or text to arrange a time for a quick check-in? Also, let us know if you have any issues or questions about the app. Looking forward to hearing from you.” Texts were accompanied by a comic-style picture of the research team, which matched the graphic style of the app.

Participants received a phone credit voucher as reimbursement for their participation and to enable ongoing contact with the research team. Research officer contact guidelines for intervention and follow-up were strictly scripted in recognition that the human-support component of digital health approaches requires definition and concurrent evaluation [23]. Following completion of all study activities, participants were then gifted the mobile device if they did not already own one (they were not advised upon entry to the study that the devices would be given to them at completion).

### Outcomes

Outcome measures were completed at baseline and 4 weeks. The primary outcome, psychological distress, was measured by the K10 [30]. The K10 is a 10-item measure of psychological distress that is sensitive to symptoms of both anxiety and depression [30]. Responses are on a 5-point Likert scale. K10 is one of the Australian Mental Health routine outcome measures and has been used in full and abbreviated forms (eg, K5, K6) in state- and nation-wide First Nations surveys [26,27,31,32]. For the period July 2012-June 2013, the Australian Mental Health Outcomes and Classification Network (AMHOCN) reports mean K10 scores for ambulatory patients with mood disorders (ie, outpatients returning to community after being treated acutely) across Australia of 27.6 (SD 8.5) upon return to community, 22.0 (SD 8.5) at a 91-day review, and 18.4 (7.6) upon discharge from outpatient service [33]. Considering these findings and those of our previous study with an Australian First Nations sample [18], a change or difference in K10 scores of 5 points would be considered clinically significant. Secondary outcome measures included the 2-question Patient Health Questionnaire (PHQ-2) with wording adapted by Brown and colleagues for First Nations Australians [34,35], the Generalized Anxiety Disorder short form (GAD-2) [35], the Leeds Dependence Questionnaire (LDQ) [36], the short form of the Alcohol Use Disorders Identification Test (AUDIT-C), the Drug Use Disorders Identification Test (DUDIT), the parent-rated Strengths and Difficulties Questionnaire (SDQ) [37], the General Help Seeking Questionnaire (GHSQ) [38], and a question adapted from the Client Service Receipt Inventory (CSRI) to determine the degree of concurrent mental health service use [39].

The PHQ-2 is a short-form version of the PHQ-9, which is designed to establish a psychiatric diagnosis of depression and has shown diagnostic, criterion, and construct validity [40,41]. The PHQ-9 has been tested in Indigenous groups and adapted to include simplified response categories [34,42,43], as well as specifically adapted for the central Australian context [34]. The 2-item PHQ-2 asks respondents to estimate the frequency of 2 symptoms (low mood and anhedonia) over the past 2 weeks on a 4-point Likert scale (0-3), with increasing scores indicating greater symptom severity. Total scores range from 0 to 6. A score of 3 or above has been shown to have a sensitivity of 83% and a specificity of 90% for the detection of major depressive disorder (MDD) [40]. The tool has also demonstrated utility for adolescent samples [44]. The GAD-2 asks respondents to estimate the frequency of 2 symptoms (nervousness and the ability to control worrying) over the past 2 weeks, with the 4 options and total scores ranging from 0 to 6 [45]. A cutoff score of 3 has been shown to have a sensitivity of 0.76 and a specificity of 0.81 [45]. The brief versions of the tools (ie, the PHQ-2 and the GAD-2) have previously been demonstrated to be reliable and valid for assessing change over time in clinical samples, with test-retest reliability of 0.79 for the PHQ-2 and 0.81 for the GAD-2 [35].

Three substance misuse screening tools were trialed. The LDQ was identified as a relatively brief (10-item) self-report measure sensitive to mild, moderate, and severe levels of dependence on alcohol and other drugs. Scores range from 0 to 30 and are intended to capture the graded intensity of the dependence syndrome. The ability to capture dependence severity simultaneously across all substance classes, including alcohol, sensitivity to change, high internal consistency (α=.93), and clinical and research utility for young adults were identified as advantages [36,46]. The AUDIT-C is a shortened version of the AUDIT [47], which is the best practice tool currently recommended for alcohol screening in the general population and performs well for adolescents [48,49] and across ethnic groups [50]. The AUDIT-C consists of 3 items to determine the risk of hazardous and harmful drinking and alcohol dependence [47]. The AUDIT-C is scored on a scale of 0-12 points (scores of 0 reflect no alcohol use in the past year), and a cutoff score of 3 or more has been proposed when used with adolescents. As the AUDIT-C only examines alcohol use, the DUDIT was also trialed as a similar, 11-item tool for identifying other drug use patterns and drug-related problems. The first 9 items are scored on a 5-point scale ranging from 0 to 4, and the last 2 are scored on a 3-point scale with values of 0, 2, and 4. Total scores range from 0 to 44, with higher scores suggesting a more severe drug problem. The cutoff score for any type of problematic use (ie, harmful use, substance abuse, or dependency) is generally recommended as 6 for men and 2 for women. The DUDIT is reported to have adequate internal consistency (α≈.9+) and test-retest reliability (ICC=0.91; Pearson *r*=0.77), with favorable sensitivity and specificity reported in a review of its psychometric properties [51].

The parent-rated SDQ was trialed as an externally rated measure to minimize participant burden. The SDQ has been used with First Nations children in national Australian surveys and consists of 25 items measuring 5 areas of psychological adjustment: emotional symptoms, conduct problems, hyperactivity, peer problems, and prosocial behavior [52,53]. Evaluation of the psychometric properties for First Nations children recommended focusing on the total difficulties score and minimizing reliance on the peer relationships subscale [53].

Demographic information was collected at baseline. App feasibility and acceptability were assessed through app use data (including number of app opens, minutes spent in the app, page visits, help accessed, etc) and qualitative interviews at 4 weeks, exploring subjective experience. App ratings (ie, from 1=“didn’t like it at all” to 5=“liked it a lot”), look and style, content, overall app rating, supportive text message check-ins, and involvement in the study were also collected to assess acceptability. Study feasibility was assessed through recruitment and retention rates. Exit interviews investigated participants’ subjective experiences, likes and dislikes in terms of content, feel, look, and style, barriers and facilitators to use, suggested improvements, and engagement with the app. They also explored participants’ experiences of being involved in the study. Interview transcripts were analyzed by 4 authors (KD, JP, MS, and JF) using a deductive approach consistent with the above categories in line with research questions [54].

Field notes were examined for further indicators of acceptability, feasibility, any adverse events, and any feedback on study measures used to aid the interpretation of the data.

Feasibility was explored through the average number of minutes used and number of app opens over the 4-week period, as well as how many had completed their own quest. Acceptability was determined through thematic analysis of subjective experiences and high app ratings (ie, mean ≥3) in each of the areas measured.

### Sample Size

For a paired-samples 2-tailed *t* test using the primary outcome K10, with a mean difference of 5 points and an SD of differences of 7, an α level of .05, and a power of 0.8, the total sample size required is 18. We anticipated a difference of at least 5 points would be indicative of clinically significant change [14,55].

### Data Analysis

Demographic information, subjective ratings, and app use data are summarized using descriptive statistics (eg, means and SDs). Paired sample *t* tests were conducted for those with complete data (ie, baseline and follow-up assessment) for each of the outcome measures, and within-group effect sizes (Cohen *d*) are reported. These were completer analyses, and there was no imputation of missing data. Exit interviews were deidentified and transcribed by an external transcribing service, then analyzed using a deductive approach to examine themes relevant to app use, likes, dislikes, facilitators and barriers to use, and suggested changes, as well as reflections on the study process. Four research team members coded the data separately and then discussed it to reach a consensus on relevant themes.

## Results

### Feasibility of Study and Outcomes

The study recruitment rate was 62.5% for those referred (35/56) and 81% for those able to be contacted (35/43) with a retention rate of 90% (30/33) for those who actually entered the study after screening.

**Figure 3:**
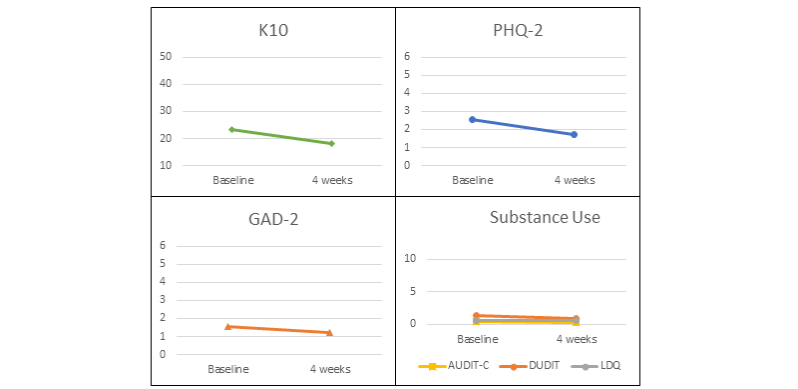
Change over time for K10, PHQ-2, GAD-2, and Substance Use Measures (AUDIT-C, DUDIT, LDQ). AUDIT-C: Alcohol Use Disorders Identification Test-Concise; DUDIT: Drug Use Disorder Identification Test; GAD-2: Generalized Anxiety Disorder; K10: Kessler Psychological Distress Scale (10-item); LDQ: Leeds Dependence Questionnaire; PHQ-2: Patient Health Questionnaire.

All mental health indicators (ie, K10, PHQ-2, and GAD-2) showed a trend toward improvement on the repeated measures *t* tests, with PHQ2 and K10 reaching clinical and statistical significance (see Table 1 and Figure 3). There was little reported substance use in this group, and no significant changes were observed for the AUDIT-C, DUDIT, or LDQ (see Table 1). The SDQ was only completed at both time points by 4 parents or guardians, and no significant differences were detected on this measure. There was little service use among the participants (see Table 2), but the most reported service used was psychologist or school counselor (n=7; 23% at baseline; n=4; 13.3% at follow-up) and Anglicare NT headspace (n=5; 16.7% at both baseline and follow-up).

In order to assess the feasibility of using a short form of the K10 (ie, the K5), the *t* test was repeated using the K5 as the dependent variable. The change remained significant (*t*_29_=5.07; *P*<.001). As shown in Table 1, mean scores at baseline were 11.97 and 9.07 at follow-up, with a large effect size (0.93).

**Table 1:** Means and *t* tests for mental health and substance use measures at baseline and 4 weeks.

Measure	Baseline, mean (SD)	4 weeks, mean (SD)	Mean (SD) difference	*t* test (*df*)	*P* value	Effect size	95% CI
Kessler Psychological Distress Scale (K10)	23.20 (7.39)	18.20 (5.20)	5.00 (5.92)	4.63 (29)	<.001	0.85	2.79 to 7.21
Patient Health Questionnaire (PHQ-2)	2.53 (1.55)	1.70 (1.29)	0.83 (1.11)	4.09 (29)	<.001	0.75	0.42 to 1.25
Generalized Anxiety Disorder short form (GAD-2)	1.53 (1.33)	1.2 (1.16)	0.33 (1.16)	1.58 (29)	.13	0.28	–0.10 to 0.77
Alcohol Use Disorders Identification Test (AUDIT-C)	0.44 (1.25)	0.26 (0.81)	0.19 (0.62)	1.55 (26)	.13	0.31	–0.06 to 0.43
Drug Use Disorders Test (DUDIT)	1.25 (2.78)	0.85 (2.77)	0.41 (1.80)	1.17 (26)	.25	0.23	–0.31 to 1.12
Leeds Dependence Questionnaire	0.57 (1.73)	0.57 (2.10)	0.00	0.0 (29)	*P*>.99	0	–0.29 to 0.29
Strengths and Difficulties Questionnaire (SDQ-parent rated)–total difficulties (n=4)	37.00 (4.16)	32.75 (3.10)	4.25 (3.95)	2.15 (3)	.12	1.07	–2.03 to 10.53

**Table 2:** Service use at baseline and follow-up.

Service used	Baseline participants, n	Follow-up participants, n
Hospital emergency department	1	0
Hospital mental health ward	1	0
Child and youth mental health service	1	0
GP^a^ or Aboriginal Community Controlled Health Service	4	1
Psychologist or school counselor	7	4
Headspace	5	5
National helpline	1	0
Other, for example, residential rehab, Clontarf	5	6

^a^GP: general practitioner.

### App Use

Descriptive statistics reflecting engagement with the app are summarized in Table 3 for 29 of the 30 participants (data missing for one participant and data not updated at follow-up for 5 participants, as phones were not available to connect to the internet, so that is likely to be a conservative estimate for 5 participants). Participants spent on average 37 minutes in the app. Three participants (10%) only opened the app once (presumably at baseline with the researcher). The app help page was accessed by 14 (47%) young people at least once, of whom one accessed the Australian Crisis Lines webpage and another accessed the Kids Helpline phone number from the app. All but 3 participants accessed their own quest to input their own story and goals, and 16 (55%) completed all 10 levels. For the levels, the most amount of time on average was spent in the fishing game on “Ramones Quest” (mean 1.30, SD 2.85) and the bubbles game on “My Quest” (mean 1.28, SD 5.98).

Separate regression analyses were conducted to see if age, time spent in the app, and number of app opens predicted mental health outcomes (K10, GAD-2, or PHQ-2). Age, use time, and number of app opens did not predict scores on any of the 3 mental health outcomes (K10, GAD-2, or PHQ-2). Simple bivariate correlations also showed no significant relationship between age and the number of app opens (*r*=0.01; *P*=.95) or number of minutes spent in the app (*r*=0.04; *P*=.85).

**Table 3:** App use statistics for sample (n=29).

	Mean (SD)	Minimum	Maximum
Minutes spent in app	37.87 (27.67)	10.33	143.28
Number of app opens	5.69 (3.78)	1	16
Number of times help accessed	0.90 (1.21)	0	5

### User Experience Ratings

Users generally rated the app positively, with mean ratings around 4 (on a scale from 1-5) for each of the 5 items: look and style, content, overall rating, check-in text messages, and study acceptability (see Table 4).

**Table 4:** Mean acceptability ratings for elements of the app and study^a^.

	Mean (SD)	Minimum	Maximum	Frequency scored 5
Q1: Look and style	4.28 (0.70)	3	5	12
Q2: Content	4.38 (0.86)	2	5	17
Q3: Overall rating	4.59 (0.50)	4	5	17
Q4: Check-ins	4.12 (0.83)	3	5	10
Q5: Study acceptability	4.62 (0.62)	3	5	20

^a^Ratings on scale of 1=“didn’t like it at all” to 5=“liked it a lot.”

### User Feedback

In terms of subjective experience, participants reported that it helped them feel relaxed, happy, calm, or safe (P23, P35, P30). It helped one reflect on their life or “check back in with yourself” (P27). Another said it helped them “feel better about myself” (P29). Participants suggested the app could help them or their mates get help in tough times. “I used it to like give me strategies and that to deal with tough times” (P35). “It was just a good experience. You need to focus on yourself sometimes and make goals for yourself. And, yeah. Especially if you’re going through a rough time and all that, and you forget people are there for you. The app says, ‘Do you have people there for you?’ And you remember. Like, ‘Oh, yeah. There is people still there for you’” (P27).

Overwhelmingly, playing the games (P5, 9, 22, 23, 24, 25, 26, 29, 31,32, 33, 35, 38) was mentioned as a favorite part of the app, as was watching the videos (P9, 11, 22, 24, 26,27, 29, 38). At least five participants liked remembering and identifying their strengths (P24, 26, 29, 38, 33). The rewards (P27, 29, 38), character stories (P1, P22, P23, P29), adding photos of family (P26) and people who care about you (P5, 35, 24, 27), and listening to the music (P26) were also mentioned as being liked.

Participants liked the look and feel of the app, it “felt natural. It wasn’t something that was like foreign” (P38). Others said it was “comfortable and homey” (P24) and “traditional” (P30). One participant suggested it “reminded me of home” (P24). Participants liked how colorful it was and the use of cultural imagery and stories “The colors are really good...and really effective. I mean the Indigenous side of it and all that” (P27). “It was nice having them talk about their culture and stuff” (P1). “I like all the little pictures” (P23).

Reasons for use included that it helped them feel relaxed, helped if they were bored, used it if their other phone was flat, checked in, and helped if they were feeling down. “I really like it because it’s helped me like get happy. Like if I was sad, or lazy, or something, I’d just look over the app and it would just get me up and going.” (P8). The text messages sent as reminders were thought to prompt use. Participants generally thought the app would be useful for First Nations young people aged 10-16 or 18 years while one or two thought it might also be useful for adults or younger kids and could even be “relatable for other people of different heritage” (P38).

Barriers to use included not having the app on their own phone (due to it only being available on Android devices for the trial), forgetting about it, its repetitiveness, and having better things to do or no time. A couple of participants reported getting lost or confused, but most reported that the app was easy to use and understand. Others suggested that not knowing what else to do once they had finished all the quests prevented them from using the app again. “So, when you completed both stories, I didn’t really know what to do after that. I kind of got stuck” (P8). While some enjoyed the games, others said they were too easy or too slow and preferred more challenging games to be included. A few participants reported that they did not really read the stories; they just skipped over them. Some did not see parts of the app, particularly the “get help” page. Some admitted that they had not used the app much or hadn’t used it again once they completed it. When they did use it, it was commonly only once or twice a week and often at night.

Suggestions were made to help overcome some of these reported barriers, which overwhelmingly included adding more content and variety, including additional characters or stories and more interactive or challenging games. The stories could include both older and younger characters, role models (eg, famous people and their stories), and more in-depth, place-specific stories. “Yeah, ‘cause it went really quick, them 2 journeys. I kept watching them and watching them over again” (P23). Other suggestions were to be able to “share your own story” (P8) or “do it for your mum or your dad as well” (P24). One participant suggested having a bit more instruction or a tutorial on how to work through the app. Additional audio was mentioned by a few participants that could be used in various ways. For example, a voice with encouraging feedback (eg, “Well done. You’re doing great” [P24]) or to read out the stories for those who have trouble or do not like reading (P35, 31). Notifications from the app were generally suggested as useful reminders to use or revisit the app, particularly after 2 weeks or when new content becomes available. One person also suggested some more customization settings would promote use, and another wanted more real-life videos (with movement rather than static imagery).

Participants often felt a sense of pride at having been involved in the project “I actually felt really good for this. Like, one of the first people to try.... Like, Holy Moley! I’m one of the first people to use the app” (P32). Most participants in the school or educational setting were happy to be interrupted in class to participate and reported that the process was good and that they felt they could say no to participating.

In summary, these results suggest that the app was thought to be acceptable, (“It’s a good app” [P1]; “I enjoyed it” [P38]; “I really like it” [P8]), easy to use (“I liked how it was really simple to do” [P35]), and culturally relevant (“it gives out like a cultural–culture pictures” [P23]; “it was nice having them talk about their culture” [P1]; “It made me feel good like that there was an app for us” [P24]) “And really effective. I mean the Indigenous side of it and all that” [P27]) and useful (“it made me feel more–felt better about myself” [P29]; “It kind of like calmed me down.... I felt like, better and sort of safer” [P30]). While it seemed that the app was not overly engaging, with some only using it once or twice, improvements were suggested that might increase engagement, including app reminders, more variety in the content, the addition of audio, more interactivity, and more stories, videos, and challenging games.

## Discussion

### Principal Results

This study assessed the feasibility, and acceptability of the newly developed AIMhi-Y app. Results demonstrated that the AIMhi-Y app is a feasible and acceptable approach to improving mental health for First Nations youth. Findings showed improved mental health outcomes for participants following 4 weeks of AIMhi-Y app use. Statistically and clinically significant improvements in psychological distress and depressive symptoms were demonstrated over the study period. However, due to the lack of appropriate control, the role the app played in these findings is unclear. These results are encouraging, as no adverse events were forthcoming and the app appeared to provide benefits. The app was also deemed acceptable by study participants in their ratings and reviews of the app. Young people reported that they found the app easy to use, culturally relevant, and useful. Engagement with the app was restricted to around 37 minutes on average, with an average of 6 opens during the 4 weeks. Suggestions were made for increasing engagement with the app that included notifications, an increased and greater variety of content, including more challenging games, and additional videos. The feasibility of the study was also demonstrated with a 62% recruitment and 90% retention rate observed and high study acceptability ratings.

### Comparison With Prior Work

The findings of significant reductions in psychological distress and depressive symptoms mirror similar recent findings in a trial of a suicide prevention app for Aboriginal and Torres Strait Islander young people, the iBobbly app [16]. iBobbly demonstrated significant improvements after 6 weeks, and our study showed significant improvements after 4 weeks. The study also demonstrated no significant deterioration in the specific domains measured. Taken together, these results provide encouraging support for the utility of these dMH apps and suggest that appropriately designed dMH apps such as these are a feasible and acceptable means of lowering symptoms for mental health disorders in regional and remote First Nations communities.

The findings also suggest that the measures used were appropriate and feasible for detecting change over time in this sample. They were well received, as demonstrated by the young people’s willingness to complete and confidence in responding, as well as their ability to demonstrate statistically significant improvements over a relatively short period. The brief versions of the tools have previously been demonstrated to be reliable and valid for assessing change over time in clinical samples [35], and this appears to hold true for our adolescent sample. Given this finding, the use of the brief versions is recommended to limit participant burden in future studies. The utility of the drug and alcohol use measures, however, was more difficult to determine in our sample of young people who demonstrated minimal substance use generally. The degree to which these measures can demonstrate change in this population over this period is, therefore, less clear. Previous research has demonstrated that changes in AUDIT-C scores over one year do reflect changes in drinking for adults and can predict future problematic drinking in adolescents [48,56]. Thus, the use of a brief assessment such as the AUDIT-C is likely to be adequate for this group. However, the SDQ may not be a feasible measure for future trials. It provided limited outcome data and was completed at both time points by only 4 parents or caregivers. Significant investment may be needed to ensure follow-up, especially for those young people living remotely or somewhat independently from their primary caregiver or parent.

Despite the positive results in well-being measures, this study also reflects previous literature suggesting that there is often high enthusiasm for dMH but low uptake and sustained use of mobile mental health apps [20]. While ratings and subjective reports indicated that the app was acceptable to participating young people, use data and subjective reports reflected modest user engagement. Time spent in the app averaged 37 minutes, and there were an average of 6 app opens per user over the 4-week period. Use was comparable to a previous study of another First Nations–specific app targeting suicide prevention (ie, iBobbly), in which participants used the app on average for 73 minutes with 12 logins on average over 6 weeks [15]. AIMhi Stay Strong MCP is a brief intervention that is the foundation for the AIMhi-Y app [18]. AIMhi Stay Strong MCP was developed to be administered over two 20-minute sessions. A recent RCT trialing AIMhi Stay Strong MCP in the form of the AIMhi Stay Strong app demonstrated significant improvements in distress and depression after 2 clinician-supported 20-minute sessions with the app [14]. While most of the app use in this study was self-directed, it is feasible that 40 minutes of use may be sufficient to elicit some change. It is also important to note that our intervention sent only one SMS message each week and that more frequent messages may have increased engagement.

In the context of other apps, this level of engagement is not unusual. Industry market research has reported that only 38% of users engage with a particular app more than 11 times, and 24% of users abandon an app after only one use [57]. Several ways of increasing user engagement have been suggested, which can include both improvements to the features of the app and the systems around them [29]. Improving app features might focus on increasing appeal, improving usability, or enhancing adherence [29]. Things like having a simple and intuitive user interface, delivering concise information in app messages, personalization and customization, incentives, updates, and new content, and good onboarding can improve user retention [57]. Others suggest offering a challenge may improve engagement over short periods, thereby allowing some therapeutic benefit and the potential for developing it into a habit [58]. In line with the above findings, participants in this study suggested including notifications, additional and greater variety of content, more challenging games, audio, and additional videos as potential ways to increase engagement.

Gamification (ie, the use of game design elements and features in nongame contexts) is commonly offered as a key opportunity to improve both appeal and adherence, which fits with the preferences of participants in this study, who favored gamification elements such as rewards, mini-games, and character stories [29,59]. Gamification features (eg, a narrative or theme, progress feedback, rewards, leaderboards, badges, customizable avatars, personalization, social interaction, competition, or user choice) need to be well designed, integrate seamlessly with the technology, and have a clear purpose and user involvement early in their design for greatest feasibility and acceptability [60,61].

Low engagement may be one of the reasons for equivocal reports when it comes to app effectiveness. If a user fails to engage with an app, it is unlikely to lead to benefits or improved outcomes. Issues with intervention engagement are not restricted to digital interventions, and similar challenges can occur with face-to-face delivery [58]. Nevertheless, the benefits of mobile apps are more consistently achieved in the context of human support [23]. Recent evidence suggests that the nature of that support is an important element in improving outcomes. Support that targets engagement alone is not sufficient to improve clinical outcomes beyond those of unsupported interventions [8]. On the other hand, support that addresses the reasons people might fail to benefit from dMH interventions, such as deficiencies in usability, engagement, fit, knowledge, or implementation, is likely to be more effective. A focus on both increasing engagement and furthering knowledge and implementation of the skills learned in the dMH intervention is needed [8]. Providing clear guidelines for how, when, and why to use support might also be of benefit [23] and highlight new research questions to be answered beyond this study.

Further co-design of the human support and implementation components of this intervention is likely to strengthen its effectiveness and is currently underway, including contextualizing it to other Australian locations given there is regional variation in First Nations cultures across Australia. The use of user-centered design that emphasizes deep engagement with key stakeholders and their organizational and social contexts has been recommended [62] and will be applied to this intervention. This is particularly important as research suggests that optimization of the design of human support services may have a greater impact on clinical outcomes than does the design of the technologies [62]. For providers (care managers, physicians, and mental health providers), the dMH tool must fit into their workflows and offer some meaningful benefit rather than just adding another task to their workdays in order to aid implementation [23]. While optimizing the human support component and considering ease of implementation are important, there is also a need to further develop the appeal of dMH interventions such as this to increase reach to the 80% of young people who are not already accessing professional treatment [63].

### Limitations

While the findings from this study appear promising, they must be considered in light of several limitations. As mentioned, the lack of a control group limits the degree to which we can attribute the outcome changes to the intervention. Future research using a hybrid trial design is recommended to investigate effectiveness and implementation success concurrently. Such a design might address the increasing evidence of a research-to-practice gap in this field of technology-enabled services (or dMH services) [23]. In addition, the study sample was relatively homogenous in that it was derived primarily from First Nations young people engaged in school and residing in an urban setting (Darwin). While we attempted to recruit a diverse sample through the inclusion of participants attending a mental health service and a residential drug rehabilitation service (from remote communities), the majority of participants recruited were through the school site, thus limiting the generalizability of the findings. On the other hand, if a greater diversity of participants from additional regions is included in future studies, it may require tailoring to context through the co-design of additional app elements or content relevant to the First Nations cultures in that region. Participants were also willing volunteers who were chosen for referral by participating key site contacts, and thus the resulting sample may have resulted in selection bias.

### Conclusions

This study showed positive effects on well-being following 4 weeks of AIMhi-Y app use. While the lack of a control group tempered the strength of these findings, the relative feasibility and acceptability of the intervention were demonstrated. High approval ratings were observed alongside modest engagement, suggesting strategies for increasing engagement, such as well-designed human support and technological and content improvements, may lead to increased use and thus increased benefit. This study supports earlier research suggesting that dMH apps that are appropriately designed with and for the target populations are a feasible and acceptable means of lowering symptoms for mental health disorders among First Nations young people.

## Abbreviations

AIMhi: Aboriginal and Islander Mental Health Initiative
AIMhi-Y: Aboriginal and Islander Mental Health Initiative for Youth
AUDIT-C: Alcohol Use Disorders Identification Test (short form)
CBT: cognitive behavioral therapy
CSRI: Client Service Receipt Inventory
dMH: digital mental health
DUDIT: Drug Use Disorders Identification Test
ERG: Expert Reference Group
GAD-2: Generalized Anxiety Disorder Scale (2-item)
GHSQ: General Help Seeking Questionnaire
IYRG: Indigenous Youth Reference Group
K10: Kessler Psychological Distress Scale (10-item)
LDQ: Leeds Dependence Questionnaire
MCP: motivational care planning
NT: Northern Territory
PHQ-2: Patient Health Questionnaire (2-item)
RCT: randomized controlled trial
SDQ: Strengths and Difficulties Questionnaire
